# A lifespan single-cell atlas of the human developing hippocampus benchmarks familial Alzheimer's disease brain organoids.

**DOI:** 10.64898/2026.09.18.752796

**Published:** 2026-09-20

**Authors:** Ekaterina Ivleva, Emil Kruikov, Joseph F. Arboleda-Velasquez, Petr Baranov

## Abstract

Familial Alzheimer's disease (fAD) is an early-onset form of AD caused by autosomal-dominant variants in APP, PSEN1, or PSEN2, with PSEN1 accounting for most genetically defined cases [1]. The hippocampus is among the earliest and most severely affected brain regions in AD [2,3]. Human induced pluripotent stem cell (iPSC)-derived brain organoids recapitulate key features of early human brain development and provide a tractable model for studying how fAD mutations perturb neurodevelopmental processes [4]. However, their interpretation is complicated by heterogeneous regional identity, variable maturation state, and cell-type composition across protocols [5,6]. Existing single-cell studies of human hippocampus cover prenatal [7] and postnatal [8-10] stages but do not provide a continuous developmental reference. By elevating the atlas approach in utilizing single-cell RNA-sequencing data, we obtain standardized information on the organoid cell class and type composition and maturation states. Here, we constructed the Human Developing Hippocampus Atlas (HuDeHA), an integrated single-cell reference comprising 658,059 cells spanning post-conceptional week 3 to 15.3 years, and used it to benchmark iPSC-derived brain organoids carrying PSEN1 E280A which is associated with fAD in a large Colombian population. Reference-based mapping revealed altered cellular composition in PSEN1 E280A organoids, including reduced radial glia and increased neural crest-derived neurons. These changes were accompanied by cross-lineage transcriptional alterations, including broad upregulation of the ventral patterning factor MEIS2 and reduced expression of the αβ-binding protein transthyretin (TTR) in choroid-plexus and ependymal-associated populations. Reconstructed neuronal-lineage trajectories showed a shift toward mature states in PSEN1 E280A organoids. Together, these findings establish HuDeHA as a resource for developmental benchmarking of hippocampus-relevant organoid systems and describe cell-lineage-specific developmental changes in PSEN1 E280A organoids that may inform interpretation of early cellular alterations in fAD.

## Full Text Availability

The license terms selected by the author(s) for this preprint version do not permit archiving in PMC. The full text is available from the preprint server.

